# Imaging interlayer exciton superfluidity in a 2D semiconductor heterostructure

**DOI:** 10.1126/sciadv.adr1772

**Published:** 2025-01-03

**Authors:** Jacob Cutshall, Fateme Mahdikhany, Anna Roche, Daniel N. Shanks, Michael R. Koehler, David G. Mandrus, Takashi Taniguchi, Kenji Watanabe, Qizhong Zhu, Brian J. LeRoy, John R. Schaibley

**Affiliations:** ^1^ Department of Physics, University of Arizona, Tucson, AZ 85721, USA.; ^2^ McCormick School of Engineering, Department of Materials Science and Engineering, Northwestern University, Evanston, IL 60208, USA.; ^3^ IAMM Diffraction Facility, Institute for Advanced Materials and Manufacturing, University of Tennessee, Knoxville, TN 37920, USA.; ^4^ Department of Materials Science and Engineering, University of Tennessee, Knoxville, TN 37996, USA, USA.; ^5^ Materials Science and Technology Division, Oak Ridge National Laboratory, Oak Ridge, TN 37831, USA.; ^6^ Department of Physics and Astronomy, University of Tennessee, Knoxville, TN 37996, USA.; ^7^ Research Center for Materials Nanoarchitectonics, National Institute for Materials Science, 1-1 Namiki, Tsukuba 305-0044, Japan.; ^8^ Research Center for Electronic and Optical Materials, National Institute for Materials Science, 1-1 Namiki, Tsukuba 305-0044, Japan.; ^9^ Guangdong Basic Research Center of Excellence for Structure and Fundamental Interactions of Matter, Guangdong Provincial Key Laboratory of Quantum Engineering and Quantum Materials, School of Physics, South China Normal University, Guangzhou 510006, China.; ^10^ Guangdong-Hong Kong Joint Laboratory of Quantum Matter, Frontier Research Institute for Physics, South China Normal University, Guangzhou 510006, China.

## Abstract

Excitons, which are Coulomb bound electron-hole pairs, are composite bosons and thus at low temperature can form a superfluid state with a single well-defined amplitude and phase. We directly image this macroscopic exciton superfluid state in an hBN-separated MoSe_2_-WSe_2_ heterostructure. At high density, we identify quasi-long-range order over the entire active area of our sample, through spatially resolved coherence measurements. By varying the exciton density and sample temperature, we map out the phase diagram of the superfluid. We observe the superfluid phase persisting to a temperature of 15 K, which is in excellent agreement with theoretical predictions. This works paves the way to realizing on chip superfluid structures capable of studying fundamental physical behaviors and quantum devices that use superfluidity.

## Teaser

A two-dimensional gas of excitons transitions into a coherent superfluid.

## INTRODUCTION

Bosons, unlike fermions, can all occupy the same quantum mechanical state. At low temperatures bosons become degenerate, forming a macroscopic wave function. While two-dimensional systems cannot have true long-range order, at sufficiently high densities bosons in two dimensions (2D) have been predicted to undergo a Berezinskii-Kosterlitz-Thouless phase transition to a superfluid state (*1*, *2*). Van der Waals heterostructures are an exciting platform for the study of these correlated states due to their wide tunability through the choice of materials and coupling between layers (*3*). In certain bilayer semiconductor systems, interlayer excitons (IXs) can form, which are composite bosons, composed of Coulomb bound electrons and holes in opposite layers (*4*). MoSe_2_-WSe_2_ semiconductor heterostructures host IXs due to their type-II band alignment (*5*). These IXs have been shown to host long lived valley polarization (*6*), highly tunable energies (*7*), rich moiré physics (*8*–*11*), and tunable quantum dots (*12*, *13*). The introduction of an hBN spacer between the MoSe_2_ and WSe_2_ layers suppresses the moiré potential and extends the lifetime of these IXs by orders of magnitude, allowing for long-range IX transport and reports of increased temporal coherence (*14*–*16*), correlated insulating states (*17*), and correlated fluids (*18*). Pioneering theoretical work by Fogler *et al.* (*19*), which predicted the existence of high-temperature IX superfluidity in an hBN-separated homobilayer, has led to intense theoretical interest in IX superfluidity (*20*–*22*). While evidence of degenerate exciton states have been reported in some 2D materials (*23*–*25*), and superfluidity in graphene (*26*), experimental demonstrations of IX superfluidity in 2D semiconductors are lacking.

## RESULTS

**Fig. 1.**
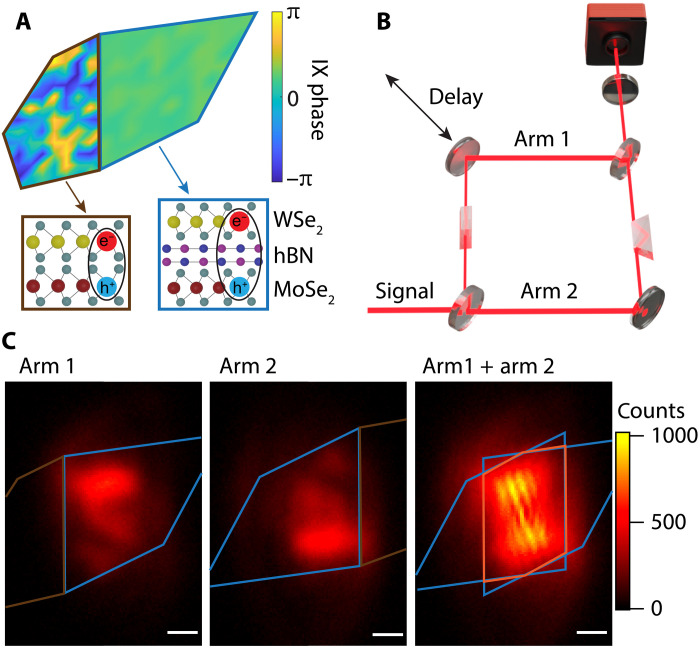
Long-range spatial coherence from interlayer excitons (IXs). (**A**) Top and side views of 2D heterostructure: monolayer MoSe_2_ (yellow and green), WSe_2_ (red and green), and bilayer hBN (pink and blue). The direct contact side (DC region) is outlined in brown, and the hBN-separated side (hBN region) in blue. The color depicts IX quantum phase. As IX density is increased the IXs in the hBN region take on a near uniform phase as they enter a superfluid state. The DC region always shows highly non uniform IX phase. (**B**) A depiction of the experimental set up. IX PL signal was sent through two arms of a Mach-Zehnder interferometer. Arm 1 and arm 2 images were inverted using two dove prisms. A delay stage was used to control the time delay of the two arms. The PL and interferogram images were measured with a cooled camera. (**C**) PL signal and interferogram from the hBN-separated region when excited at 150 μW at 1.6 K. The hBN region and rotated hBN regions are outlined in blue, the overlap region between the two arms is outlined in orange. White scale bars represent 1 μm.

In this work, we image IX superfluidity in an hBN-encapsulated MoSe_2_-bilayer hBN-WSe_2_ heterostructure. The geometry of the sample is shown in Fig. 1A. One region is constructed with the MoSe_2_ directly contacting (denoted DC) the WSe_2_ with a near 0° twist angle. In the other region, a bilayer hBN spacer (denoted hBN), was inserted between the MoSe_2_ and WSe_2_ layers to suppress the moiré potential and extend the IX lifetime to 1.9 μs (fig. S1). Here, we study the IXs that were optically excited in the hBN-separated region. Specifically, we performed spatial coherence measurements on the photoluminescence (PL) emitted from the hBN-separated region as a measure of the quasi-long-range spatial coherence of the IX phase.

A depiction of the experiment is shown in Fig. 1B. The sample was held in an optical cryostat at temperatures down to 1.6 K. An integrated high–numerical aperture (NA) objective lens was used to excite the sample with a 720-nm laser and to collect the PL emitted by the IXs. The collected PL was sent through a Mach-Zehnder interferometer. Dove prisms were introduced in the interferometer arms so that the PL image in one arm was rotated 180° from the other to allow for nonlocal spatial coherence measurements (Fig. 1C). A delay stage was used to control the time delay between the arms. The PL in each arm was recombined and then imaged onto a camera resulting in the interferogram shown in Fig. 1C.

The resulting interferogram (Fig. 1C) is given by (*27*, *28*)

$$I_{t}\left(\vec{r}\right)=I_{1}\left(\vec{r}\right)+I_{2}\left(\vec{r}\right)+2\sqrt{I_{1}\left(\vec{r}\right)I_{2}\left(\vec{r}\right)}g^{\left(1\right)}\left(\vec{r},t\right)$$ (1)

Where $I_{t}\left(\vec{r}\right)$ is the measured PL intensity on the camera at position $\vec{r}$, $I_{1}$, and $I_{2}$ are intensities from arm 1 and arm 2 of the interferometer respectively (Fig. 1C), and $g^{\left(1\right)}\left(\vec{r},t\right)$ is the first-order coherence function at position $\vec{r}$ and time delay t. For short time scans near-zero time delay, $t_{0}$ (<20 fs), there is no appreciable temporal decay in $g^{\left(1\right)}$ (fig. S2A). Therefore, the temporal portion of the coherence function oscillates with time delay as $g^{\left(1\right)}\left(\vec{r},t\right)$ = $g^{\left(1\right)}\left(\vec{r}\right)\text{cos}\left[\frac{2\pi }{\lambda }c\left(t-t_{0}\right)+\vec{q}\cdot \vec{r}+\phi \left(\vec{r}\right)\right]$ where $\vec{q}=\frac{2\pi \alpha }{\lambda }$ is the spatial frequency of the fringes associated with a tilt angle α in one of the arms and ϕ is the phase of the wave function. This allows both the spatial dependence of the amplitude and phase to be extracted. We define the interference contrast at a given time delay as S$(\vec{r},t):$

$$S\left(\vec{r},t\right)=\frac{I_{t}\left(\vec{r}\right)-I_{1}\left(\vec{r}\right)-I_{2}\left(\vec{r}\right)}{2\sqrt{I_{1}\left(\vec{r}\right)I_{2}\left(\vec{r}\right)}}=g^{\left(1\right)}\left(\vec{r}\right)\text{cos}\left[\frac{2\pi }{\lambda }c\left(t-t_{0}\right)+\vec{q}\cdot \vec{r}+\phi \left(\vec{r}\right)\right]$$ (2)

**Fig. 2.**
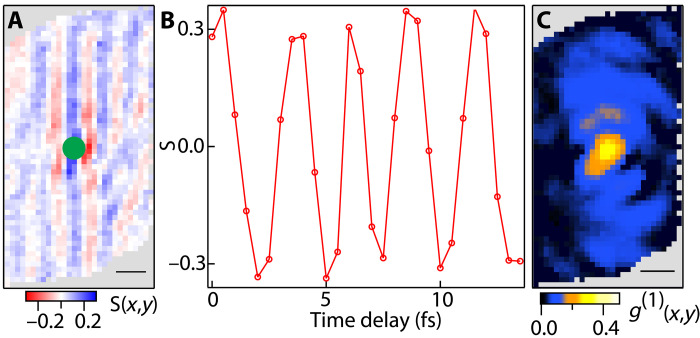
Extracting the spatial coherence function $g^{(1)}(\vec{r})$ from the interferograms. (**A**) Coherence fringes, S, resolved over the overlap region outlined in Fig. 1C by subtracting the arm 1 and arm 2 contributions from the interference pattern shown in Fig. 1C and normalizing. (**B**) Intensity oscillation at the location of the green pixel when scanning the delay stage in 0.5-fs steps over 13 fs. (**C**) Spatially resolved first-order coherence $g^{\left(1\right)}\left(\vec{r}\right)$ measured by taking the amplitude of the oscillation shown in (B) for all pixels. The area outside the overlap region is shown in gray. Scale bars, 500 nm.

The observed interference contrast (from the interferogram shown in Fig. 1C) is shown in Fig. 2A. A mask is applied to show only the overlap region of the interfered PL. A series of delay stage scans were taken over 13 fs with 0.5-fs steps centered around $t_{0}$. Figure 2B shows the interference contrast for a single point (green dot in Fig. 2A) as a function of time delay. The amplitude of the interference contrast for each point ($\vec{r}$) is used to extract the spatial coherence function $g^{\left(1\right)}\left(\vec{r}\right)$ (text S1).

Figure 2C shows the amplitude of the first-order spatial coherence function for the hBN-separated emission at 1.6 K and 150 μW excitation power. The two arms of the interferometer are rotated 180° relative to each other about the center of the sample region. The resulting interferogram is therefore measuring the PL emitted from point $\vec{r\prime }$ from the sample interfering with PL from point $-\vec{r\prime }$, where $\vec{r\prime }$ is measured from the center of the sample region. As expected, a small central spot of high coherence is observed (corresponding to $\vec{r\prime }=-\vec{r\prime }=0$) with a diameter on the order of the resolution (point spread function) of the objective (~0.5 μm) (*29*), while a lower but finite degree of coherence is present over the entire overlap region of the arm 1 and arm 2 PL. The 0.5-μm–diameter central spot persists for all powers and temperatures and is a result of the spatial coherence of the same point in space with itself.

**Fig. 3.**
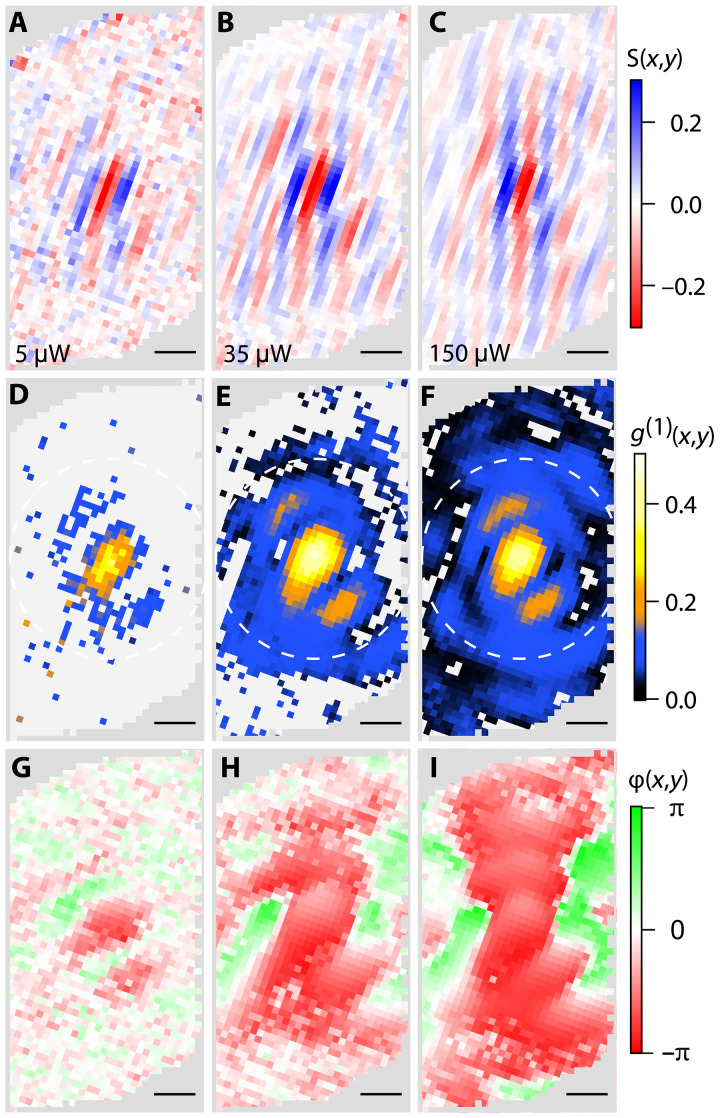
Power dependence of the spatial coherence 15 K. (**A** to **C**) Power-dependent, averaged fringe contrast from the hBN-separated emission. As the power (IX density) is increased, fringes spread across the length of the entire overlap region. (**D** to **F**) Power-dependent spatial coherence of IX emission in the hBN-separated device. A bright central spot is observed at all powers with a diameter of ~0.5 μm, the resolution of the collection objective. At higher powers, longer-range spatial coherence is observed further away from the central spot. The white dashed circle shows the ring radially averaged over to produce the phase diagram in Fig. 4E. (**G** to **I**) Power-dependent phase resolved for the hBN-separated region. As coherence spreads, nearly uniform phase is observed across the entire overlap region.

Berezinskii-Kosterlitz-Thouless (BKT) theory predicts that when a system of IXs with a density $n_{\text{IX}}$ cools below a critical temperature given by $T_{\text{BKT}}\approx 1.3\frac{\hbar^{2}n_{\text{IX}}}{k_{B}m_{\text{IX}}}$ (*19*, *30*), they will undergo a phase transition to a macroscopic superfluid state (where, ℏ is the reduced Planck’s constant, $k_{B}$ is Boltzmann’s constant, and $m_{\text{IX}}$ is the IX mass). The transition temperature of this state increases linearly with increasing density and is categorized by quasi-long-range coherence with a near-uniform phase. In the experiment, the IX density is controlled by varying the excitation laser power (text S3). Figure 3 (A to C) shows the excitation power dependence of the interference fringes at 15 K. At low excitation power (Fig. 3A), there are only interference fringes at the center of the image due to the self-coherence of the central spot. There is no long-range coherence as the IXs are in the gas phase. As the excitation power is increased, the interference fringes spread over the entire overlap region demonstrating the emergence of quasi-long-range coherence in the system and the transition to a superfluid state. Figure 3 (D to F) shows the amplitude of the spatially resolved first-order coherence for the fringes shown in panels (A) to (C). As the excitation power is increased, the spatial extent of the (blue) long-range coherence increases and becomes nearly uniform across the overlap region (Fig. 3F). Figure 3 (G to I) shows the phase for each power, showing that the coherence fringes maintain a uniform phase over the entire hBN overlap region (text S2). This is contrasted with coherence measurements performed on the DC region (fig. S3), which show a strong central $g^{\left(1\right)}$ spot in agreement with a previous work (*28*). In addition, the DC region shows clear dislocations in the fringe contrast and a nonuniform amplitude and phase.

**Fig. 4.**
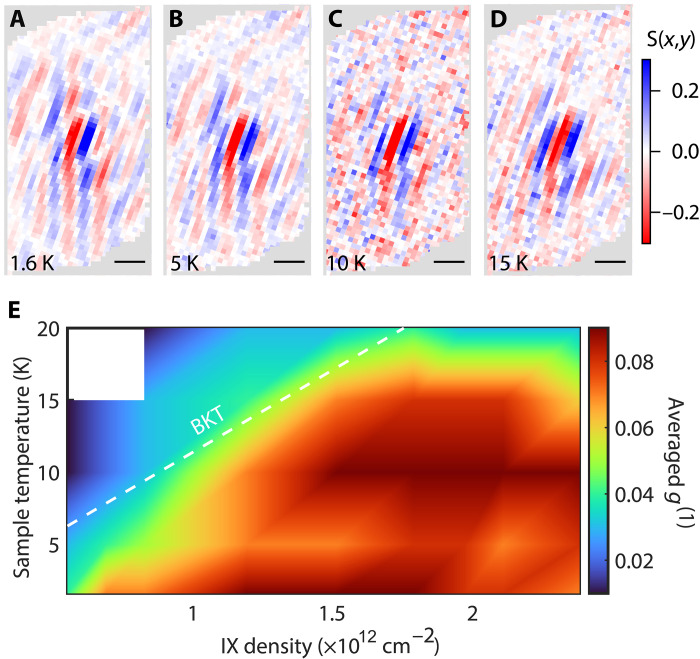
IX transition to a superfluid phase. (**A** to **D**) Fringe contrast within the overlap region for four different sample temperatures at 10-μW excitation. As temperature is increased long-range coherence decreases. (**E**) Phase diagram resolved by radially averaging over the ring shown in Fig. 3 (D to F) as a function of IX density and sample temperature. As the temperature reaches 20 K, the signal to noise becomes too low for low-excitation powers, so the two lowest powers are excluded from analysis. The white line shows the theoretically predicted BKT line from reference (*19*).

To understand the temperature and density dependence of the IX coherence, a series of measurements was taken for excitation powers from 5 to 300 μW at temperatures of 1.6, 5, 10, 15, and 20 K. The temperature dependence of the interference fringes in the hBN-separated region for 10 μW excitation power is shown in Fig. 4 (A to D). For this fixed exciton density, the spatial extent of the interference fringes abruptly decreases between 5 and 10 K, showing the predicted loss of the coherent superfluid state as the temperature is increased. We note that the results were reproducible over multiple measurements and cooldowns.

Because of the unknown interlayer carrier transfer rate in a bilayer hBN-separated heterostructure, estimation of the IX density for the hBN region is difficult. However, extensive research has been done on direct contact heterostructures (*31*, *32*), allowing for an estimation of density in the DC region. Since the interlayer carrier transfer rate in direct contact heterostructures is much faster than the intralayer decay rate, the exciton density is determined by the lifetime of IXs. Using the known lifetimes of IXs in each region at low power, and the measured intensities of each region’s PL, the relative densities can be estimated, and the density of the hBN region can be resolved. For each excitation power, the IX density was calculated using the measured PL intensity and lifetime (text S3 and fig. S6).

To quantify the density and temperature dependence of the quasi-long-range spatial coherence, the first-order coherence function was radially averaged 1.2 μm from the central spot (white dashed line in Fig. 3, D to F) corresponding to the coherence between all locations 2.4 μm apart from each other on the sample. This location was selected as it is well away from the central spot but the PL signal remains nearly constant (figs. S4 and S5). This average value is plotted for each power (density) and temperature in the phase diagram (Fig. 4E). In the hBN-separated region, an abrupt transition temperature is observed below which quasi-long-range coherence is observed. This transition agrees very well with the predicted BKT transition shown by the dashed white line. We note that the phase diagram in the DC region is featureless (see fig. S7), indicating the absence of a transition to the superfluid state. We note that lower value of $g^{\left(1\right)}$ measured on the hBN-separated region is dominated by interferometer noise arising from the longer integration time required to obtain sufficient signal to noise (see text S4 and fig. S8).

A feature of a superfluid state is algebraic scaling in both temporal coherence and spatial coherence (*33*). Figure S9A shows a power law fit of the form $g\left(u\right)=Au^{b}$ to the radially averaged spatial coherence away from the center and the temporal coherence at 1.6 K for an IX density of ~2 × 10^12^ cm^−2^. The higher-temperature trace in fig. S9B shows a faster decay, which is expected as the IXs is no longer in a superfluid state. Furthermore, fig. S10 shows that there is substantial narrowing of the IX PL emission with increasing excitation laser power.

## DISCUSSION

Previous studies of correlated excitonic fluids could not perform direct optical measurements like the methods used here due to the limited IX PL signal emitted by >1-nm–thick hBN-separated MoSe_2_-WSe_2_ heterostructures (*18*, *23*). This work presents, to our knowledge, the first direct observation of the quasi-long-range spatial coherence present in an IX superfluid, persisting to temperatures of 15 K. Here, we observe clear onsets of spatial coherence at low temperature and high density in excellent quantitative agreement with BKT theory (*19*).

Beyond this work, we foresee that IX superfluids will be of immense interest in qubit and transistor design, where superfluid IXs can enable lossless IX transport carrying valleytronic information (*34*), and can mimic the physical behaviors of superconducting circuits (*35*, *36*). The direct observation of IX superfluidity presented here allows for the construction of on-chip, experimentally accessible temperature qubits, and IX superfluid circuitry using 2D semiconductors.

## MATERIALS AND METHODS

### Sample fabrication

All 2D material layers were isolated by exfoliation onto Si/SiO_2_ wafers using scotch tape. MoSe_2_ and WSe_2_ monolayers were selected with atomic force microscopy and optical contrast. Polarization resolved second-harmonic generation was used to align the MoSe_2_ and WSe_2_ layers to near 0° (*37*, *38*), and a bilayer hBN layer was chosen to separate the MoSe_2_ and WSe_2_ layers. The optical signatures of the device are consistent with other R-type heterostructures (*10*). In addition to extending the lifetime of IXs, the bilayer hBN spacer suppresses the moiré potential in the device (*39*). Eight-nanometer–thick hBN was used as the top layer, and 22-nm–thick bottom hBN was used. The layers were stacked using a polymer-based dry transfer method (*40*).

### Optical measurements

Spatial PL measurements were taken by exciting the sample in an optical cryostat with a 76-MHz, 120-fs Ti:sapphire laser centered about 720 nm [see fig. S2 (B to E) for coherence measurements of the laser]. PL images were rotated using two dove prisms. The sample’s temperature was controlled by an attocube attodry 2100 cryostat. The sample was excited and imaged using an integrated NA = 0.81 objective lens. The hBN region emits PL centered about 1.42 eV (870 nm) and the DC region about 1.34 eV (920 nm) (*10*). Longpass filters (800 and 850 nm) were used to reject the 720-nm laser and monolayer exciton PL. When imaging the hBN-separated signal, a 900-nm shortpass filter was used to block the direct contact signal. When imaging the direct contact signal, a 900-nm longpass filter was used to reject the hBN-separated signal. The PL was magnified and imaged using a 1-m lens and a cooled scientific camera (Andor Newton). Lifetime measurements were taken using an acousto-optic-modulator–based pulse picker to increase the repetition period to 3.1 μs, and the IX PL was measured using a spectrometer and a time-correlated single-photon counting setup (Picoquant).

## Supplementary Materials

### The PDF file includes:

Supplementary Text Figs. S1 to S10 References
