# Supplementary material for: Imaging interlayer exciton superfluidity in a 2D semiconductor heterostructure
Source: Sci Adv. 2025 Jan 3;11(1):eadr1772. doi: 10.1126/sciadv.adr1772 (PMC11698081; doi:10.1126/sciadv.adr1772)
Supplement: Supplementary file 1 — Supplementary Text Figs. S1 to S10 References [file sciadv.adr1772_sm.pdf]

Science Advances
AAAS

Supplementary Materials for

Imaging interlayer exciton superfluidity in a 2D semiconductor heterostructure

Jacob Cutshall *et al.*

Corresponding author: John R. Schaibley, johnschaibley@arizona.edu

Sci. Adv. **11**, eadr1772 (2025)
DOI: 10.1126/sciadv.adr1772

This PDF file includes:

Supplementary Text
Figs. S1 to S10
References

Supplementary Text

S1. Measurement of $g^{(1)}$ Amplitude

To measure the amplitude of the spatial coherence function, interferograms were measured over a series of time delays using 0.5 fs steps. Then for each spatial location in the interferogram, the interference contrast signal was determined. Taking a temporal Fourier transform gives a signal with a component at $\omega = \frac{\lambda}{c}$. The amplitude of this component gives the amplitude of $g^{(1)}$. For spatial locations where the amplitude of $g^{(1)}$ was less than 4 times the noise floor from the Fourier transform, the value was set to 0. Due to a crack in the hBN region of the sample, the region of non-zero (blue) coherence observed in Fig. 2C is slightly narrower than the overlap region. For further analysis of the coherence signal only this non-zero region was utilized.

S2. Measurement of $g^{(1)}$ Phase

From the interferograms it is possible to extract phase information of the spatial coherence function. This is done using the following algorithm. First, we can write the interference contrast in complex exponential form,

$$S(\vec{r}, t) = g(\vec{r})\mathrm{e}^{2\pi \mathrm{i}\vec{q}\cdot\vec{r}} + g^{*}(\vec{r})\mathrm{e}^{-2\pi \mathrm{i}\vec{q}\cdot\vec{r}}.$$

where $g(\vec{r}, t) = \frac{1}{2} g^{(1)}(\vec{r})\mathrm{e}^{\mathrm{i}\left(\frac{2\pi c}{\lambda}\Delta t + \phi(\vec{r})\right)}$ and $\vec{q}$ is the vector associated with the tilt between the two arms of the interferometer which causes the spatial interference fringes. At a given time delay, the spatial Fourier transform of the interference contrast can be written as

$$\tilde{S}(\vec{k}, t) = \tilde{g}(\vec{k} - \vec{q}, t) + \widetilde{g^{*}}(\vec{k} + \vec{q}, t).$$

where $\tilde{g}$ is the Fourier transform of g. As the interference contrast is real, we can take only one of the two components of $\tilde{S}(\vec{k}, t)$ without the loss of any information. This component is then

shifted in frequency space by an amount $\vec{q}$ to obtain $\tilde{g}(\vec{k}, t)$. Lastly, we take the inverse Fourier transform to obtain the phase information as

$$\phi(\vec{r}, t) + \frac{2\pi c}{\lambda} \Delta t = \tan^{-1} \frac{\Im\, g(\vec{r}, t)}{\Re\, g(\vec{r}, t)}.$$

As the time delay is changed between each image, there is an overall phase shift of the signal due to the $\frac{2\pi c}{\lambda} \Delta t$ term. Therefore, we set the center pixel at each time delay to a fixed phase value and measure changes in phase with respect to this value. After setting the center pixel to a fixed phase, a total of 27 fringe contrast images are averaged over a time delay of 13 fs to obtain the images in Fig. 3A-C and Fig. 4A-D. The measured phase (Fig. 3G-I) is determined by averaging over the phase at each time delay.

S3. IX Density Calculation

The number of intralayer excitons and IXs can be found from the following rate equations,

$$\frac{\partial N_m}{\partial t} = \frac{\alpha\, P}{\hbar\omega} - \gamma_m N_m - \gamma_t N_m,$$

$$\frac{\partial N_{IX}}{\partial t} = \gamma_t N_m - \gamma_{IX} N_{IX},$$

where N_m is the number of excitons generated in the $MoSe_2$ layer, N_{IX} is the number of IXs in the heterostructure, γ_{IX} is the IX decay rate, γ_m is the $MoSe_2$ intralayer exciton decay rate, α is the absorption coefficient, P is the excitation power, $\hbar\omega$ is the laser photon energy, and γ_t is the $MoSe_2$-WSe_2 hole transfer rate. In the steady state, there is no time dependence in the number of excitons giving:

$$N_m = \frac{\alpha\, P}{(\gamma_m + \gamma_t)\hbar\omega},$$

$$N_{IX} = \frac{\gamma_t \alpha\, P}{\gamma_{IX}(\gamma_m + \gamma_t)\hbar\omega}.$$

To estimate the IX density from the PL measurements, the signal per unit time, calculated by summing the background subtracted PL emission on the CCD for the hBN separated and DC regions separately, can be written as:

$$I_{DC} = \frac{\beta N_{DC}}{\tau_{DC}},$$

$$I_{hBN} = \frac{\beta N_{hBN}}{\tau_{hBN}}.$$

where N_{DC} and N_{hBN} are the number of IXs in the DC and hBN regions respectively, τ_{DC} and τ_{hBN} are the IX lifetimes in the DC and hBN regions respectively, and β is a constant that accounts for loss through collection efficiency, reflection efficiency, and transmission efficiency through all optics.

At low excitation power the lifetimes of IXs in the hBN and DC regions are known (Fig. S2). γ_{IX} is found through lifetime measurements to be (40 ns)$^{-1}$ in the DC region, γ_m is estimated to be (0.5 ps)$^{-1}$ (*31*), and γ_t is taken to be (0.05 ps)$^{-1}$ (*41*) which means that N_{DC} can be simplified to:

$$N_{DC} \approx \frac{\alpha\, P\tau_{DC}}{\hbar\omega}.$$

which gives a value of β based on all measured quantities. This value of β is then utilized to calculate the number of IXs in the hBN region based on the strength of the PL signal and the measured lifetime. Using this method, the exact values of the $MoSe_2$-WSe_2 hole transfer rate and intralayer exciton decay rate do not need to be known to determine the number of IXs in the hBN region. The densities can then be calculated by dividing the number of IXs by the area which is ~4x4 μm for both the DC and hBN regions. As the excitation power is increased the density of excitons is scaled by the increase in PL intensity in both regions. These density estimates assume a constant IX lifetime with power and temperature. Due to the long IX lifetimes of the hBN and DC regions relative to the laser repetition period of 13.2 ns, power and temperature dependent

lifetimes cannot be measured under the conditions used in this experiment. However, any consequential change in lifetime should be detectable in the IX emission intensity. Fig. S6A shows minimal variation in PL intensity with temperature and an increase in emission intensity with excitation power.

S4. Analysis of Interferometer Contrast and Exposure Time

The longer exposure time (120 s) required to measure the emission from the hBN separated region of the device compared to the DC region (30 s) reduces the interference contrast and the value of $g^{(1)}$, which is due to instability of our interferometer. We can demonstrate this effect using a narrow linewidth continuous wave (CW) laser, which is a highly coherent source and should have interference contrast between 1 and -1. Figure R4 shows the interference contrast of a CW laser emitting 720 nm light taken at 30 s and 120 s exposure times. There is a clear decrease in contrast when the fringes are averaged over longer exposure times. This decrease can be quantified by fitting to the fringes with a sine wave and extracting the $g^{(1)}$ value. We find that the reported $g^{(1)}$ value decreases by 30 percent. As shown in Fig. 2 and Fig. S3, the central $g^{(1)}$ in the hBN region reaches a maximum of 0.52 and in the DC region it reaches a maximum of 0.53. We take the ratio $g^{(1)}_{max}/g^{(1)}_{laser}$ for both the hBN and DC regions to see that the hBN region reaches 85.2% of the maximum value for its exposure time, while the DC region reaches 60.9% of the maximum value at that exposure time. Once correcting for the loss of contrast due to exposure time, the hBN region shows higher coherence than the DC region.

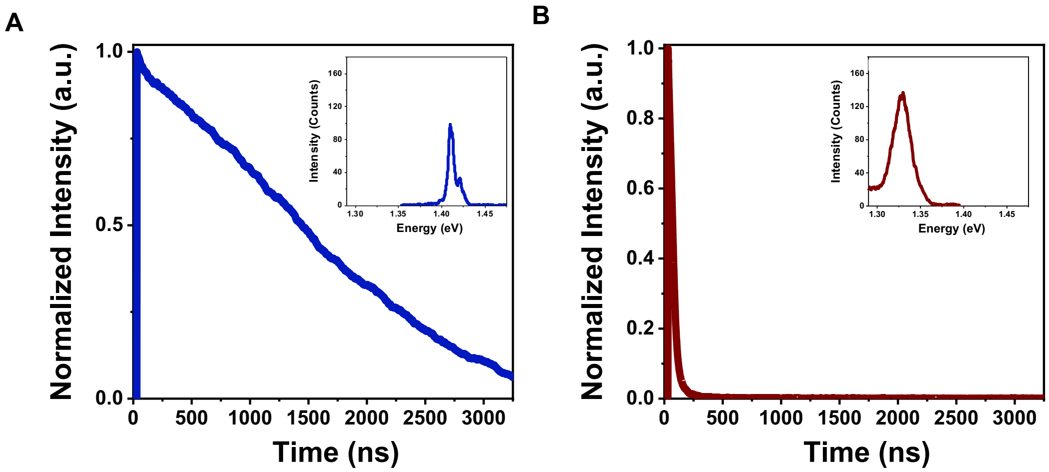

Fig. S1. IX lifetime measurements in hBN and DC regions. (**A**) The blue curve shows PL intensity as a function of time for the hBN region showing hBN IX lifetime to be ~1900 ns. (**B**) The maroon curve shows the PL intensity as a function of time for DC region showing DC IX lifetime to be ~42 ns. Both measurements were taken with 0.63 μW average excitation power. Insets show the PL spectrum of each region.

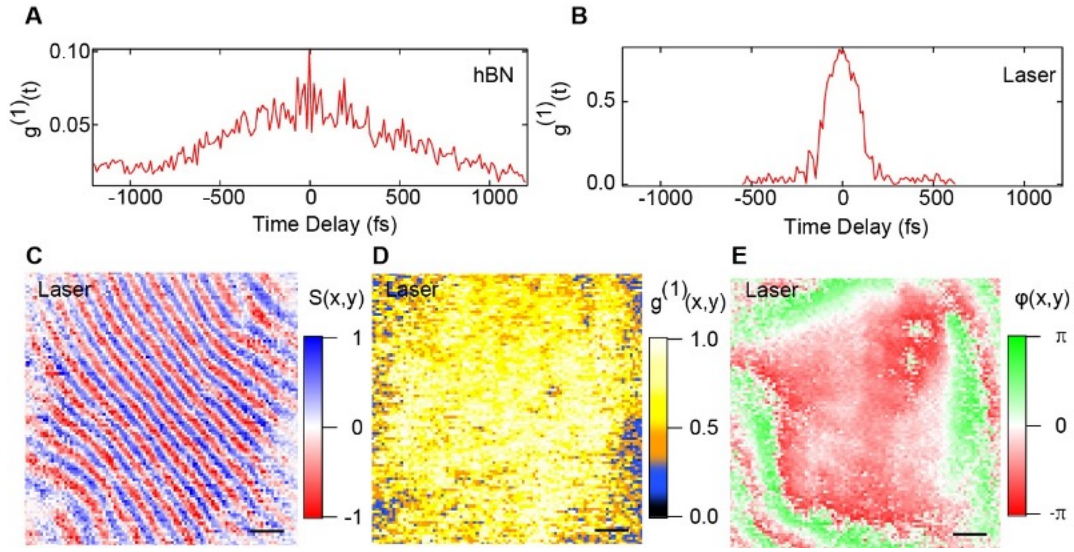

Fig. S2. Temporal coherence of hBN IX and coherence of laser. (**A**) Temporally resolved first order coherence function for the hBN region. $g^{(1)}(t)$ is resolved by taking a time scan similar to that described in Fig. 2 over 2600 fs with 13 fs steps. For each time step the amplitude of $g^{(1)}$ at the center of the image is determined. (**B**) $g^{(1)}(t)$ resolved for the laser showing a narrower temporal coherence length than the hBN separated region. (**C-E**) Fringe contrast, spatial coherence function, and phase for the laser showing the expected results for a coherent source. Scale bars represent 500nm.

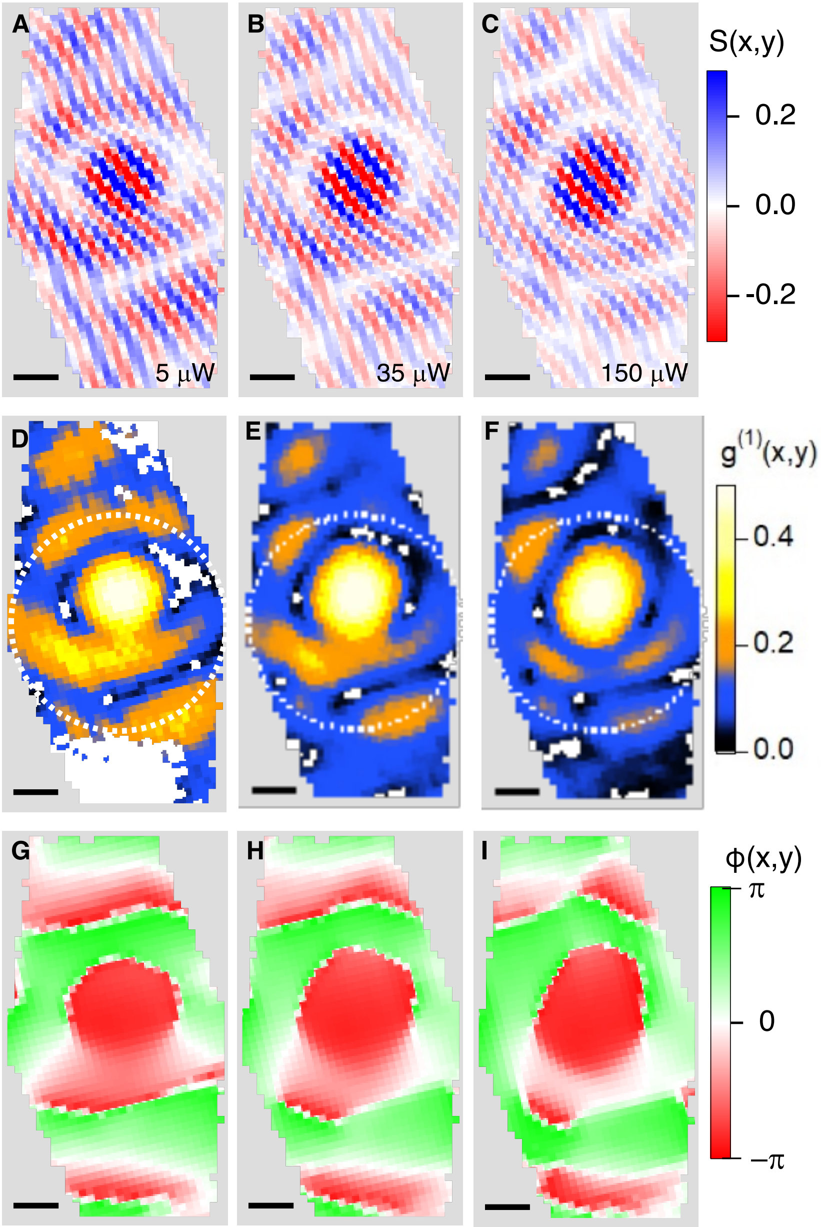

Fig. S3. Fringe contrast, spatial coherence, and phase for DC PL. (**A-C**) Fringe contrast at different excitation powers measured for the DC region of the device showing multiple discontinuities in the interference fringes over the overlap region. (**D-F**) Spatially resolved first order coherence function for the DC region showing multiple maxima and minima resulting from the discontinuities shown in A-C. (**G-I**) Fringe phase for the DC region showing non-uniform phase throughout the overlap region.

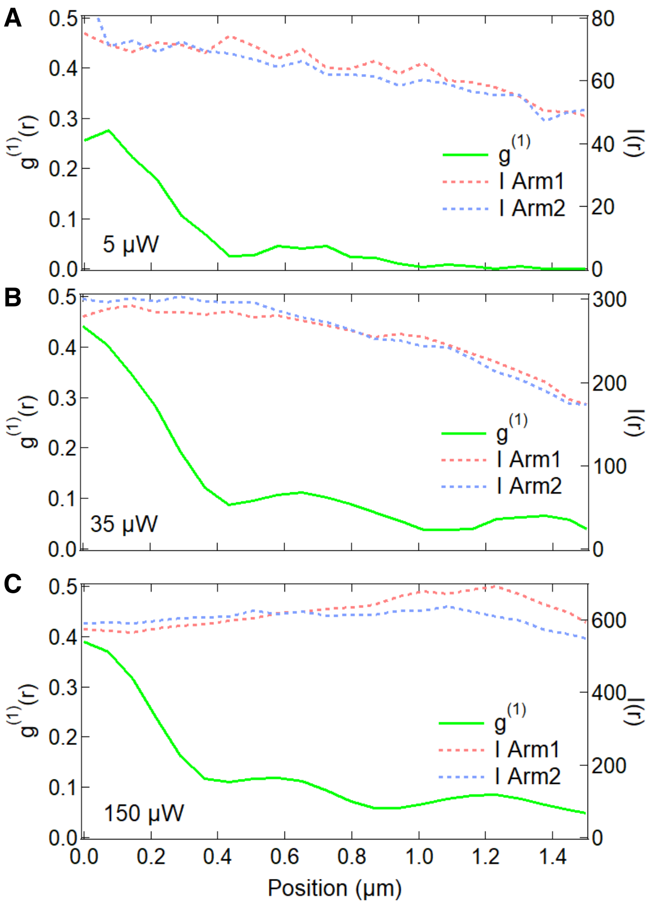

Fig S4. Radially averaged $g^{(1)}(\vec{r})$ and PL emission from hBN region at 5, 35, and 150 μW. (**A-C**) The green curves are the radially averaged first order spatial coherence function as a function of power (left axis). The dashed red and blue lines (right axis) show the radially averaged PL intensities for each arm (Arm 1 and Arm 2) of the interferometer which are nearly constant.

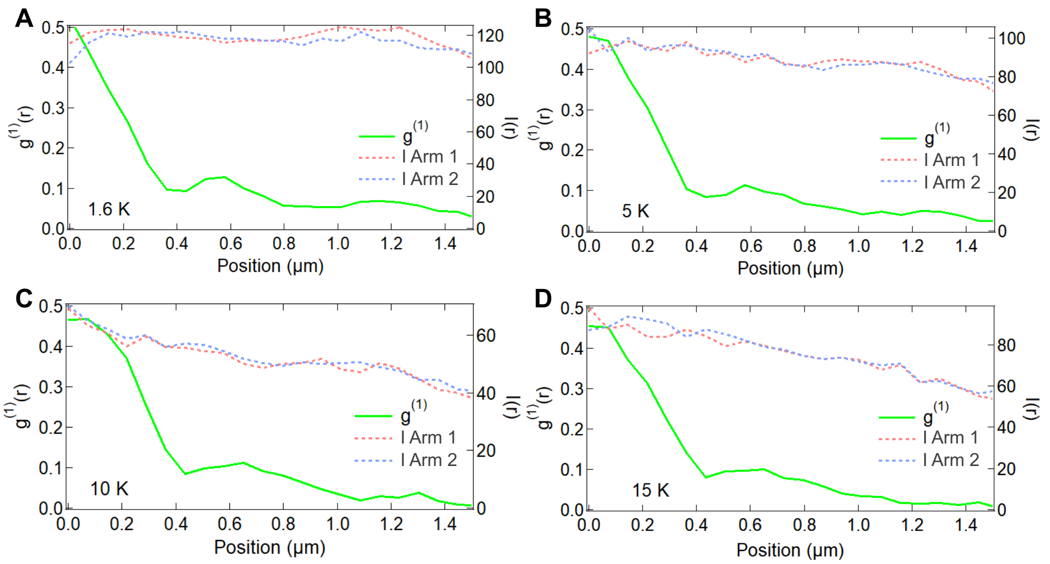

Fig. S5. Temperature dependence of radially averaged $g^{(1)}(\vec{r})$ and PL emission from hBN at 10 μW excitation. (**A-D**) The green curves show the radially averaged first order spatial coherence function for four different temperatures (left axis). The dashed red and blue lines (right axis) show the radially averaged PL intensities for each arm (Arm 1 and Arm 2) of the interferometer which are nearly constant.

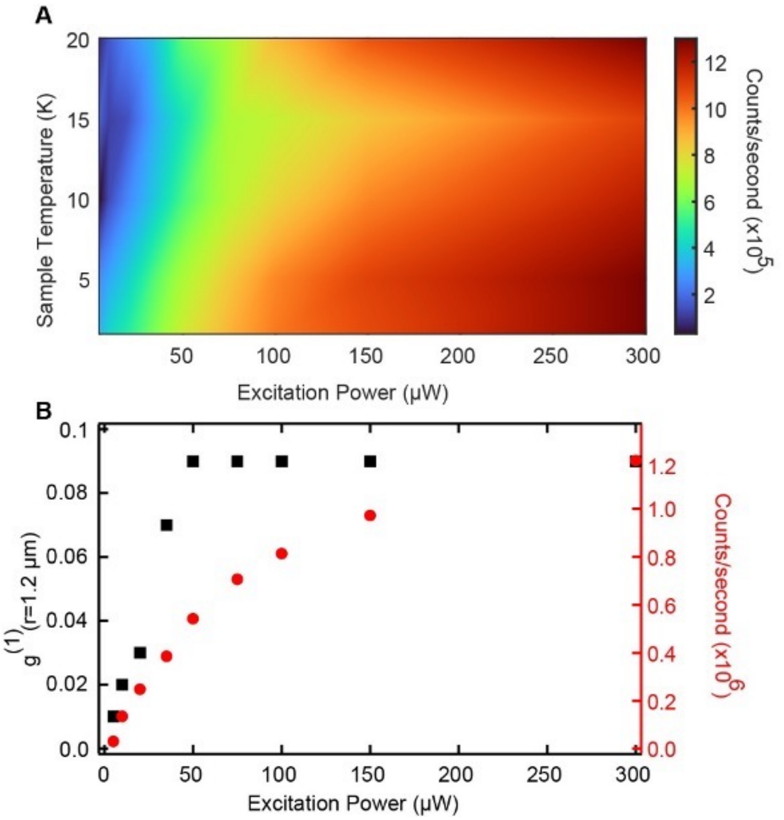

Fig. S6. Analysis of PL intensity power and temperature dependence. (**A**) Counts/second from the hBN separated IX emission integrated over the entire CCD as a function of excitation power and sample temperature showing minimal variation with temperature and increase with power. (**B**) Excitation power dependent radially averaged $g^{(1)}$ at r = 1.2 μm showing a sharp increase at low powers and constant value at high powers while emission intensity saturates at high power but continues to increase.

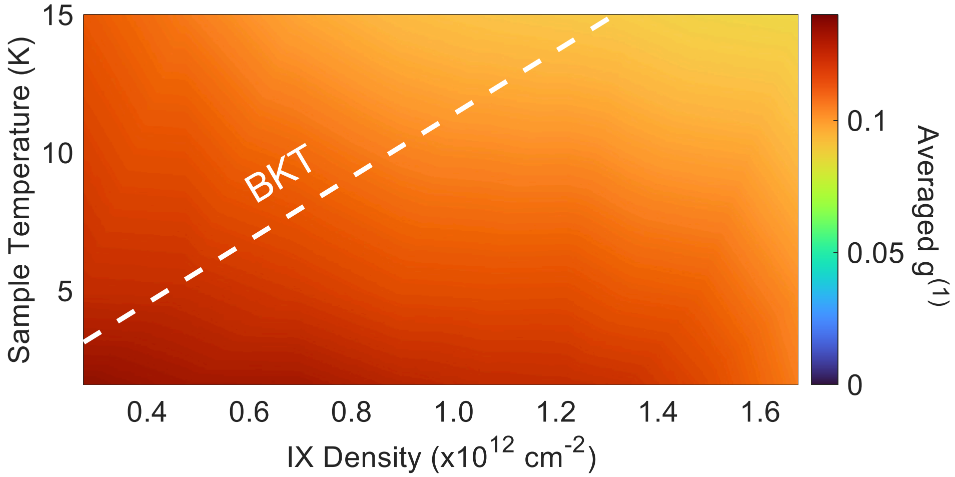

Fig. S7. Phase diagram for the DC region. The phase diagram for the DC region resolved by radially averaging over the white dashed line shown in Extended Data Fig. 3D-F. No transition is observed. The white line shows the theoretical BKT line from reference (*19*).

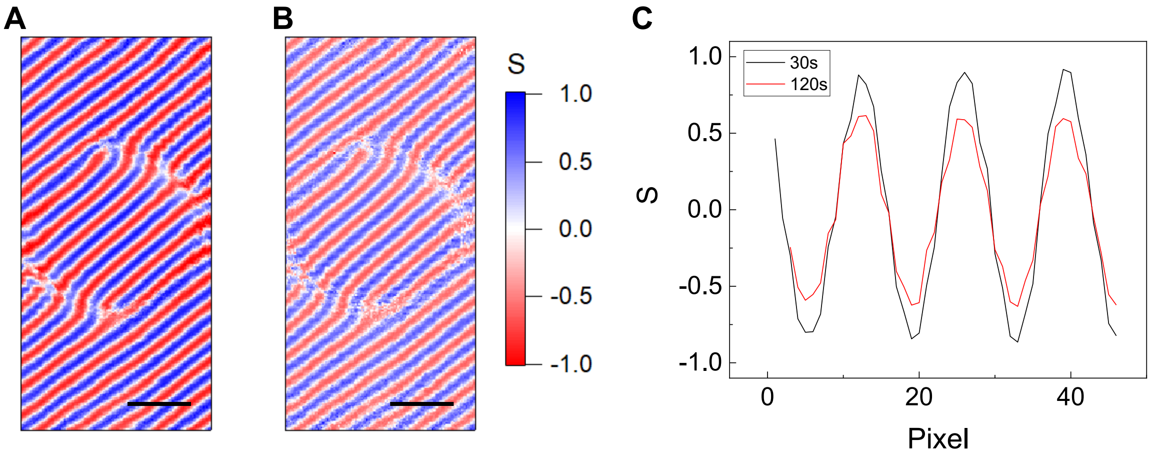

Fig. S8. Comparison of fringe contrast at different exposure times. Laser fringe interference contrast taken at (**A)** 30 s exposure time and (**B)** 120 s exposure time. (**C)** Line cuts taken perpendicular to the fringes shown in A and B.

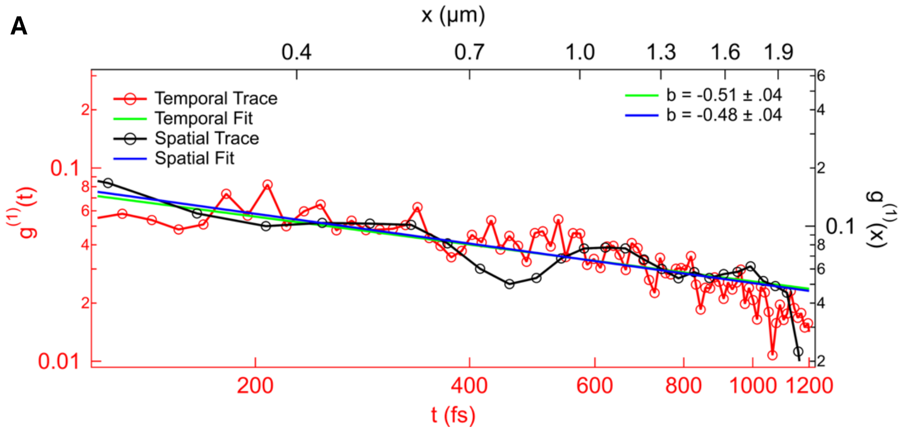

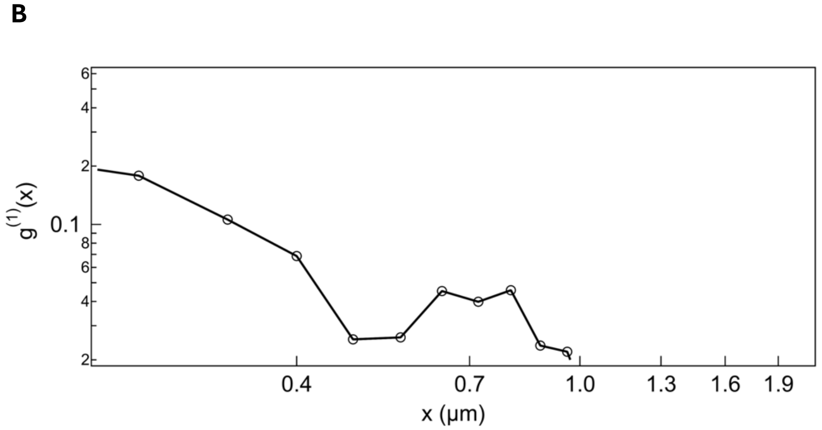

Fig. S9. **Comparison of temporal and spatial decay. (A)** Temporal coherence trace taken at 200 μW and spatial coherence trace taken at 150 μW with a power law fit of the form $g^1(u) = Au^b$, where u is either time or position, showing comparable exponents. (**B)** Spatial coherence trace at 15 K and 5 μW showing notably faster decay than the trace shown in A.

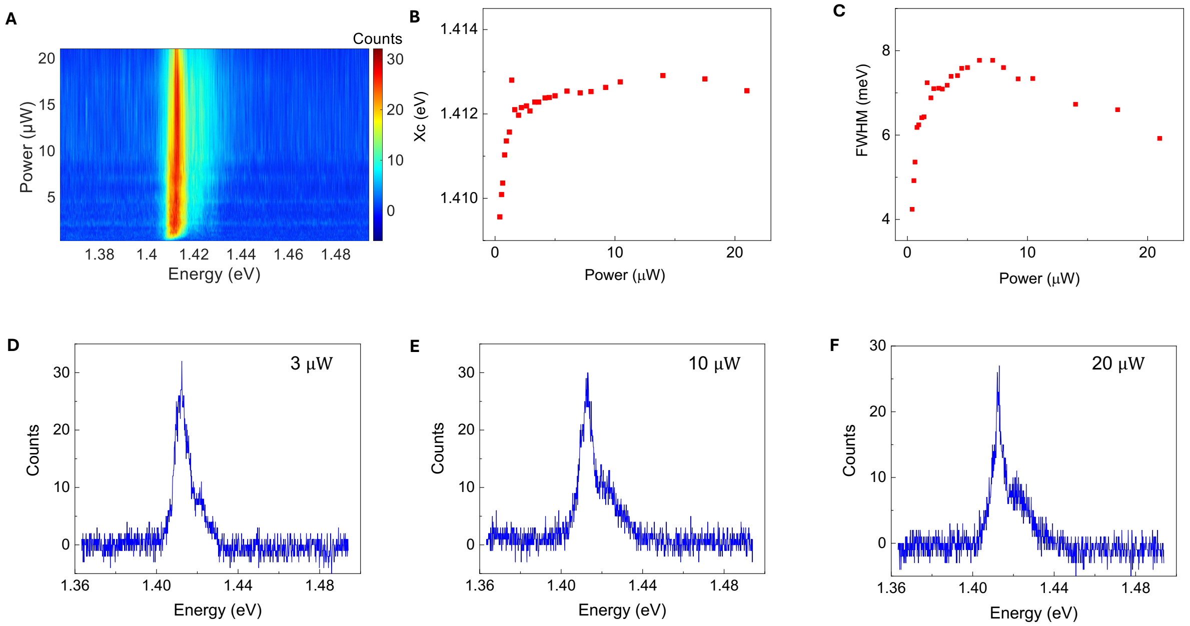

Fig. S10. Analysis of PL spectra as a function of excitation power. (A) Excitation power dependent PL emission for the hBN separated region at 1.6 K showing notable narrowing at higher powers. **(B)** Power dependent center energy showing an initial increase with excitation power. **(C)** Power dependent PL linewidth showing an initial increase followed by a decrease. **(D-F)** PL emission taken at 3 μW (D), 10 μW (E), and 20 μW showing two different peak structures at low and high densities.

REFERENCES AND NOTES

1. L. V. Keldysh, A. N. Kozlov, Collective properties of excitons in semiconductors. *J. Exp. Theor. Phys.* **27**, 521 (1968).

2. J. M. Kosterlitz, D. J. Thouless, Ordering, metastability and phase transitions in two-dimensional systems. *J. Phys. C Solid State Phys.* **6**, 1181–1203 (1973).

3. N. P. Wilson, W. Yao, J. Shan, X. Xu, Excitons and emergent quantum phenomena in stacked 2D semiconductors. *Nature* **599**, 383–392 (2021).

4. L. V. Butov, A. Zrenner, G. Abstreiter, G. Böhm, G. Weimann, Condensation of indirect excitons in coupled AlAs/GaAs quantum wells. *Phys. Rev. Lett.* **73**, 304–307 (1994).

5. P. Rivera, J. R. Schaibley, A. M. Jones, J. S. Ross, S. Wu, G. Aivazian, P. Klement, K. Seyler, G. Clark, N. J. Ghimire, J. Yan, D. G. Mandrus, W. Yao, X. Xu, Observation of long-lived interlayer excitons in monolayer $MoSe_2$–WSe_2 heterostructures. *Nat. Commun.* **6**, 6242 (2015).

6. P. Rivera, K. L. Seyler, H. Yu, J. R. Schaibley, J. Yan, D. G. Mandrus, W. Yao, X. Xu, Valley-polarized exciton dynamics in a 2D semiconductor heterostructure. *Science* **351**, 688–691 (2016).

7. L. A. Jauregui, A. Y. Joe, K. Pistunova, D. S. Wild, A. A. High, Y. Zhou, G. Scuri, K. De Greve, A. Sushko, C.-H. Yu, T. Taniguchi, K. Watanabe, D. J. Needleman, M. D. Lukin, H. Park, P. Kim, Electrical control of interlayer exciton dynamics in atomically thin heterostructures. *Science* **366**, 870–875 (2019).

8. K. L. Seyler, P. Rivera, H. Yu, N. P. Wilson, E. L. Ray, D. G. Mandrus, J. Yan, W. Yao, X. Xu, Signatures of moiré-trapped valley excitons in $MoSe_2$/WSe_2 heterobilayers. *Nature* **567**, 66–70 (2019).

9. T. Song, Q.-C. Sun, E. Anderson, C. Wang, J. Qian, T. Taniguchi, K. Watanabe, M. A. McGuire, R. Stöhr, D. Xiao, T. Cao, J. Wrachtrup, X. Xu, Direct visualization of magnetic domains and moiré magnetism in twisted 2D magnets. *Science* **374**, 1140–1144 (2021).

10. F. Mahdikhanysarvejahany, D. N. Shanks, C. Muccianti, B. H. Badada, I. Idi, A. Alfrey, S. Raglow, M. R. Koehler, D. G. Mandrus, T. Taniguchi, K. Watanabe, O. L. A. Monti, H. Yu, B. J. LeRoy, J. R. Schaibley, Temperature dependent moiré trapping of interlayer excitons in MoSe2-WSe2 heterostructures. *NPJ 2D Mater. Appl.* **5**, 67 (2021).

11. H. Liu, J. Wang, S. Chen, Z. Sun, H. Xu, Y. Han, C. Wang, H. Liu, L. Huang, J. Luo, D. Liu, Direct visualization of dark interlayer exciton transport in moiré superlattices. *Nano Lett.* **24**, 339–346 (2024).

12. D. N. Shanks, F. Mahdikhanysarvejahany, C. Muccianti, A. Alfrey, M. R. Koehler, D. G. Mandrus, T. Taniguchi, K. Watanabe, H. Yu, B. J. LeRoy, J. R. Schaibley, Nanoscale trapping of interlayer excitons in a 2D semiconductor heterostructure. *Nano Lett.* **21**, 5641–5647 (2021).

13. D. N. Shanks, F. Mahdikhanysarvejahany, M. R. Koehler, D. G. Mandrus, T. Taniguchi, K. Watanabe, B. J. LeRoy, J. R. Schaibley, Single-exciton trapping in an electrostatically defined two-dimensional semiconductor quantum dot. *Phys. Rev. B* **106**, L201401 (2022).

14. F. Tagarelli, E. Lopriore, D. Erkensten, R. Perea-Causín, S. Brem, J. Hagel, Z. Sun, G. Pasquale, K. Watanabe, T. Taniguchi, E. Malic, A. Kis, Electrical control of hybrid exciton transport in a van der Waals heterostructure. *Nat. Photonics* **17**, 615–621 (2023).

15. D. N. Shanks, F. Mahdikhanysarvejahany, T. G. Stanfill, M. R. Koehler, D. G. Mandrus, T. Taniguchi, K. Watanabe, B. J. LeRoy, J. R. Schaibley, Interlayer exciton diode and transistor. *Nano Lett.* **22**, 6599–6605 (2022).

16. L. H. Fowler-Gerace, Z. Zhou, E. A. Szwed, D. J. Choksy, L. V. Butov, Transport and localization of indirect excitons in a van der Waals heterostructure. *Nat. Photonics* **18**, 823–828 (2024).

17. L. Ma, P. X. Nguyen, Z. Wang, Y. Zeng, K. Watanabe, T. Taniguchi, A. H. MacDonald, K. F. Mak, J. Shan, Strongly correlated excitonic insulator in atomic double layers. *Nature* **598**, 585–589 (2021).

18. R. Qi, A. Y. Joe, Z. Zhang, Y. Zeng, T. Zheng, Q. Feng, J. Xie, E. Regan, Z. Lu, T. Taniguchi, K. Watanabe, S. Tongay, M. F. Crommie, A. H. MacDonald, F. Wang, Thermodynamic behavior of correlated electron-hole fluids in van der Waals heterostructures. *Nat. Commun.* **14**, 8264 (2023).

19. M. M. Fogler, L. V. Butov, K. S. Novoselov, High-temperature superfluidity with indirect excitons in van der Waals heterostructures. *Nat. Commun.* **5**, 4555 (2014).

20. F.-C. Wu, F. Xue, A. H. MacDonald, Theory of two-dimensional spatially indirect equilibrium exciton condensates. *Phys. Rev. B* **92**, 165121 (2015).

21. C. Lagoin, F. Dubin, Key role of the moiré potential for the quasicondensation of interlayer excitons in van der Waals heterostructures. *Phys. Rev. B* **103**, L041406 (2021).

22. B. Remez, N. R. Cooper, Leaky exciton condensates in transition metal dichalcogenide moiré bilayers. *Phys. Rev. Res.* **4**, L022042 (2022).

23. Z. Wang, D. A. Rhodes, K. Watanabe, T. Taniguchi, J. C. Hone, J. Shan, K. F. Mak, Evidence of high-temperature exciton condensation in two-dimensional atomic double layers. *Nature* **574**, 76–80 (2019).

24. B. Sun, W. Zhao, T. Palomaki, Z. Fei, E. Runburg, P. Malinowski, X. Huang, J. Cenker, Y.-T. Cui, J.-H. Chu, X. Xu, S. S. Ataei, D. Varsano, M. Palummo, E. Molinari, M. Rontani, D. H. Cobden, Evidence for equilibrium exciton condensation in monolayer WTe_2. *Nat. Phys.* **18**, 94–99 (2022).

25. L. Sigl, F. Sigger, F. Kronowetter, J. Kiemle, J. Klein, K. Watanabe, T. Taniguchi, J. J. Finley, U. Wurstbauer, A. W. Holleitner, Signatures of a degenerate many-body state of interlayer excitons in a van der Waals heterostack. *Phys. Rev. Res.* **2**, 042044 (2020).

26. J. I. A. Li, T. Taniguchi, K. Watanabe, J. Hone, C. R. Dean, Excitonic superfluid phase in double bilayer graphene. *Nat. Phys.* **13**, 751–755 (2017).

27. E. Y. Paik, L. Zhang, G. W. Burg, R. Gogna, E. Tutuc, H. Deng, Interlayer exciton laser of extended spatial coherence in atomically thin heterostructures. *Nature* **576**, 80–84 (2019).

28. M. Troue, J. Figueiredo, L. Sigl, C. Paspalides, M. Katzer, T. Taniguchi, K. Watanabe, M. Selig, A. Knorr, U. Wurstbauer, A. W. Holleitner, Extended spatial coherence of interlayer excitons in $MoSe_2/WSe_2$ heterobilayers. *Phys. Rev. Lett.* **131**, 036902 (2023).

29. M. M. Fogler, S. Yang, A. T. Hammack, L. V. Butov, A. C. Gossard, Effect of spatial resolution on the estimates of the coherence length of excitons in quantum wells. *Phys. Rev. B* **78**, 035411 (2008).

30. A. Filinov, N. V. Prokof'ev, M. Bonitz, Berezinskii-Kosterlitz-Thouless transition in two-dimensional dipole systems. *Phys. Rev. Lett.* **105**, 070401 (2010).

31. X. Hong, J. Kim, S.-F. Shi, Y. Zhang, C. Jin, Y. Sun, S. Tongay, J. Wu, Y. Zhang, F. Wang, Ultrafast charge transfer in atomically thin MoS_2/WS_2 heterostructures. *Nat. Nanotechnol.* **9**, 682–686 (2014).

32. C. Jin, E. Y. Ma, O. Karni, E. C. Regan, F. Wang, T. F. Heinz, Ultrafast dynamics in van der Waals heterostructures. *Nat. Nanotechnol.* **13**, 994–1003 (2018).

33. D. Caputo, D. Ballarini, G. Dagvadorj, C. Sánchez Muñoz, M. De Giorgi, L. Dominici, K. West, L. N. Pfeiffer, G. Gigli, F. P. Laussy, Marzena H. Szymańska, D. Sanvitto, Topological order and thermal equilibrium in polariton condensates. *Nat. Mater.* **17**, 145–151 (2018).

34. J. R. Schaibley, H. Yu, G. Clark, P. Rivera, J. S. Ross, K. L. Seyler, W. Yao, X. Xu, Valleytronics in 2D materials. *Nat. Rev. Mater.* **1**, 16055 (2016).

35. E. Schwartz, B. Li, A. A. Kovalev, Superfluid spin transistor. *Phys. Rev. Res.* **4**, 023236 (2022).

36. L. Amico, D. Aghamalyan, F. Auksztol, H. Crepaz, R. Dumke, L. C. Kwek, Superfluid qubit systems with ring shaped optical lattices. *Sci. Rep.* **4**, 4298 (2014).

37. L. M. Malard, T. V. Alencar, A. P. M. Barboza, K. F. Mak, A. M. de Paula, Observation of intense second harmonic generation from MoS_2 atomic crystals. *Phys. Rev. B* **87**, 201401 (2013).

38. N. Kumar, S. Najmaei, Q. Cui, F. Ceballos, P. M. Ajayan, J. Lou, H. Zhao, Second harmonic microscopy of monolayer MoS_2. *Phys. Rev. B* **87**, 161403 (2013).

39. F. Mahdikhanysarvejahany, D. N. Shanks, M. Klein, Q. Wang, M. R. Koehler, D. G. Mandrus, T. Taniguchi, K. Watanabe, O. L. A. Monti, B. J. LeRoy, J. R. Schaibley, Localized interlayer excitons in MoSe2–WSe2 heterostructures without a moiré potential. *Nat. Commun.* **13**, 5354 (2022).

40. P. J. Zomer, M. H. D. Guimarães, J. C. Brant, N. Tombros, B. J. van Wees, Fast pick up technique for high quality heterostructures of bilayer graphene and hexagonal boron nitride. *Appl. Phys. Lett.* **105**, (2014).

41. G. Moody, C. Kavir Dass, K. Hao, C.-H. Chen, L.-J. Li, A. Singh, K. Tran, G. Clark, X. Xu, G. Berghäuser, E. Malic, A. Knorr, X. Li, Intrinsic homogeneous linewidth and broadening mechanisms of excitons in monolayer transition metal dichalcogenides. *Nat. Commun.* **6**, 8315 (2015).
